# Optical Coherence Tomography in Inflammatory and Neoplastic Lesions Deforming the Choroidal Profile

**DOI:** 10.3390/diagnostics13121991

**Published:** 2023-06-07

**Authors:** Elena Bolletta, Luca De Simone, Marco Pellegrini, Chiara Preziosa, Valentina Mastrofilippo, Chantal Adani, Pietro Gentile, Fabrizio Gozzi, Luca Cimino

**Affiliations:** 1 Ocular Immunology Unit, Azienda USL-IRCCS, 42123 Reggio Emilia, Italy; elena.bolletta@ausl.re.it (E.B.); luca.desimone@ausl.re.it (L.D.S.); valentina.mastrofilippo@ausl.re.it (V.M.); chantal.adani@ausl.re.it (C.A.); pietro.gentile@ausl.re.it (P.G.); fabrizio.gozzi@ausl.re.it (F.G.); 2 Department of Biomedical and Clinical Science “Luigi Sacco”, Luigi Sacco Hospital, University of Milan, 20157 Milan, Italy; mar.pellegrini@gmail.com (M.P.); preziosachiara@gmail.com (C.P.); 3 Clinical and Experimental Medicine PhD Program, University of Modena and Reggio Emilia, 41124 Modena, Italy; 4 Department of Surgery, Medicine, Dentistry and Morphological Sciences, with Interest in Transplants, Oncology and Regenerative Medicine, University of Modena and Reggio Emilia, 41124 Modena, Italy

**Keywords:** choroid, choroidal granuloma, uveitis, tumor, optical coherence tomography, enhanced depth imaging, swept-source, OCT, EDI-OCT, SS-OCT

## Abstract

The choroid is the main part of the uvea, the vascular layer of the eye that lies between the retina and the sclera. The high vascular component of the choroid makes this structure susceptible to inflammation in multisystemic diseases, as well as the most common site of metastasis in the eye. Therefore, the choroid is involved in many pathological conditions, from uveitis to intraocular tumors. Differentiating between inflammatory and neoplastic lesions deforming the choroidal profile can sometimes be challenging. In addition, scleral disorders can also deform the choroidal profile. Choroidal imaging includes ophthalmic ultrasonography, indocyanine green angiography, and optical coherence tomography (OCT). Recent advances in choroidal imaging techniques, such as enhanced depth imaging optical coherence tomography (EDI-OCT) and swept-source optical coherence tomography (SS-OCT), have facilitated an in-depth analysis of the choroid. The purpose of this review article is to report on and highlight the most common OCT findings to help in the differential diagnosis between inflammatory and neoplastic lesions deforming the choroidal profile.

## 1. Introduction

The choroid is the largest and most posterior portion of the uvea, the vascular layer of the eye that lies between the retina and the sclera that nourishes the retinal pigment epithelium (RPE) and the outer retina. The high vascular component of the choroid makes this structure susceptible to inflammation in multisystemic diseases, as well as the most common site of metastasis in the eye [1].

The inaccessibility of the choroidal structure to direct clinical examination makes clinicians rely on imaging techniques for its evaluation. Traditional choroidal imaging modalities include indocyanine green angiography (ICGA) and ocular ultrasonography (US) [2]. More recently, the non-invasive optical coherence tomography (OCT) has been introduced. OCT uses the principle of low coherence interferometry to obtain in-depth information from various retinal structures to create cross-sectional images [2].

Conventional spectral-domain OCT (SD-OCT) has some limitations, especially in the evaluation of deeper structures such as the choroid. Enhanced depth imaging OCT (EDI-OCT) uses a scanning position closer to the eye to create an inverted SD-OCT image with the advantage of better depth sensitivity.

Swept-source OCT (SS-OCT) utilizes longer waves (>1000 nm) and has many advantages, such as higher scanning speed, reduced sensitivity loss, deeper penetration into the tissues, and better penetration across lens opacifications and hemorrhages.

The aim of this review article is to highlight choroidal imaging features using OCT that may assist in the differentiation between inflammatory and neoplastic lesions deforming the choroidal profile.

## 2. Inflammatory Lesions

### 2.1. Choroidal Inflammatory Lesions

The most frequent choroidal inflammatory diseases deforming the choroidal profile are described according to uveitis epidemiology [3].

#### 2.1.1. Acute Uveitic Phase of Vogt–Koyanagi–Harada (VKH) Disease

**Figure 1:**
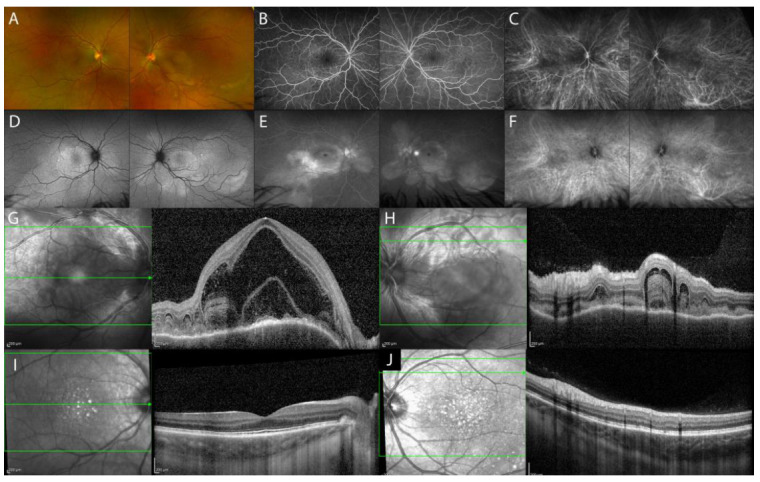
Multimodal imaging (MMI) of the acute uveitic phase of VKH disease. Ultra-wide field (UWF) pseudocolor fundus image (Optos PLC, Dunfermline, Scotland, UK) with multifocal ERD (**A**). FA showing peripapillary and posterior pole hyperfluorescence, indicating pooling of the ERD and hyperfluorescence of the optic disc (**B**,**E**). Fundus autofluorescence imaging (FAF) highlighting the ERD (**D**). ICGA hyperfluorescence with leakage from the choroidal vessels and multiple hypofluorescent dots (**C**,**F**). EDI-OCT (Spectralis OCT, Heidelberg Engineering, Heidelberg, Germany) showing multifocal ERD (**G**), RPE undulation (**H**), and diffuse thickening of the choroid, which all reduce post-treatment (**I**,**J**).

The acute uveitic phase of VKH disease is characterized by multifocal exudative retinal detachments (ERD) of the neurosensory retina secondary to diffuse granulomatous choroiditis (Figure 1A). In addition, the initial stage of the disease can present optic disc hyperemia and swelling, associated or alone [4].

Fluorescein angiography (FA) features indicate secondary retinal involvement as a consequence of severe choroiditis, including subretinal fluid pooling due to ERD and optic disc hyperfluorescence (Figure 1B,E). ICGA reveals signs of choroiditis characterized by the presence of hypofluorescent dark dots and vasculitis of choroidal vessels (fuzziness of the vessels) (Figure 1C,F) [5]. B-scan US of the eye displays ERD with associated diffuse low-to-medium reflective thickening of the choroid posteriorly [6].

In acute VKH disease associated uveitis, OCT shows ERD with subretinal fluid compartments separated by the typical septa (Figure 1G). Tsuboi et al. have described choroidal folds, which may be the result of a thickened choroid compressing Bruch’s membrane and RPE causing undulations of the RPE (Figure 1H). Choroidal folds were quantified using the RPE undulation index [7].

OCT can also be helpful in the early recognition of the predominantly optic disc swelling variant, which carries a worse prognosis [8]. In the acute phase of VKH disease, EDI-OCT reveals a markedly diffuse thickening of the choroid. Choroidal thickness may serve as a marker for the degree of choroidal inflammation, which gradually decreases with treatment (Figure 1I,J) [9]. SS-OCT provides better resolution images of the choroid than EDI-OCT does, resulting in more measurable images. When both SS-OCT and EDI-OCT images are readable, the measurements are comparable [10].

#### Chronic Recurrent Uveitic Phase of VKH Disease

Initial-onset acute VKH disease is characterized by early posterior uveitis, which, if not properly controlled, can be followed by the chronic recurrent phase of the disease with anterior segment inflammation [4]. Using ICGA, Bacsal et al. and Takemoto et al. showed concomitant subclinical choroidal inflammation in patients with anterior segment recurrence [11,12]. Choroidal thickness increases during the anterior uveitis attack, then decreases when the inflammation has resolved after treatment [12]. Instead, choroidal vascularity index (CVI) decreases during the anterior uveitis attack, returning to baseline values after treatment [13].

**Figure 2:**
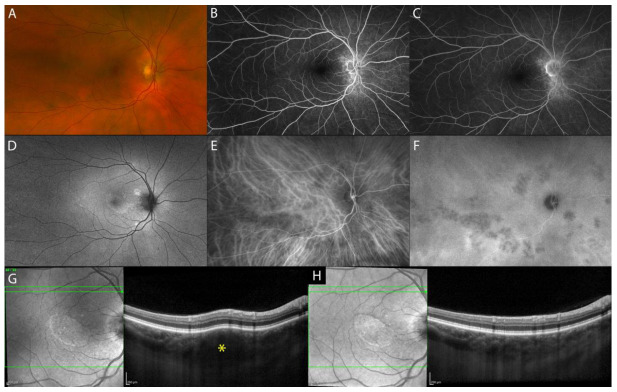
MMI of the chronic recurrent uveitic phase of VKH disease. UWF fundus image (**A**). FA (**B**,**C**). FAF (**D**). ICGA with multiple coalescent hypofluorescent dots (**E**,**F**). EDI-OCT showing corresponding localized thickening of the choroid, which assumes a convex appearance with consequent bulging of the outer retina (‘choroidal bulging’), indicated by the asterisk (**G**) and reduction post-treatment (**H**).

Sakata et al. reported a unique finding in VKH patients in the non-acute uveitic stage, “choroidal bulging”, which seems to be related to active posterior segment inflammation. It is characterized by a localized thickening of the choroid, which assumes a convex appearance with consequent bulging of the RPE/Bruch’s membrane reflective complex anterior (Figure 2) [14].

During the convalescent stage in patients with long-standing VKH disease, the choroid thickness turned out to be progressively thinner on EDI-OCT than that of normal individuals [15].

#### 2.1.2. Isolated Tubercular and Sarcoid Choroid Granulomas

Tuberculosis (TB) and sarcoidosis are chronic multisystem granulomatous diseases that share similar pulmonary and extrapulmonary manifestations, including choroidal involvement in uveitis [16,17]. The prevalence of TB uveitis (TBU) is estimated to be between 9% and 11% in endemic countries and between 1% and 6% in non-endemic countries [18]. Choroiditis is its most frequent manifestation [19]. Choroidal involvement in TBU can involve the choriocapillaris in serpiginous-like and ampiginous choroiditis or the stromal choroid in cases of choroidal granuloma, recently termed “tuberculoma” by the Collaborative Ocular Tuberculosis Study (COTS) group [20].

Ocular sarcoidosis shows a wide range of clinical manifestations, including uveitis, and can precede systemic involvement in 12–30% of cases [21]. Isolated choroidal granuloma, which can be the first manifestation of the disease, is a relatively rare lesion that occurs in approximately 5% of cases of sarcoid uveitis [22].

Both tuberculoma and isolated sarcoid granuloma may clinically simulate intraocular malignancy or other inflammatory conditions [23,24].

**Figure 3:**
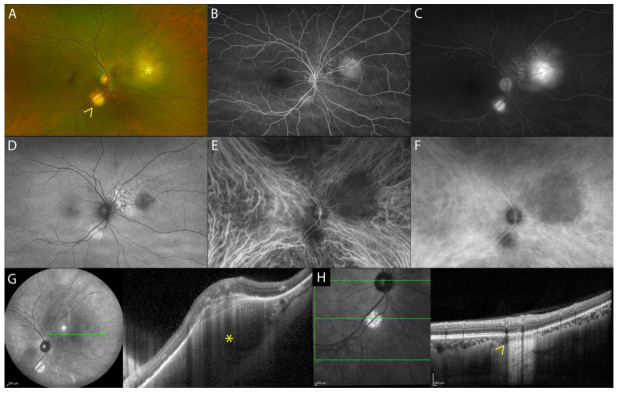
MMI of choroidal tuberculoma. UWF fundus image and FAF showing a solitary, active round-shaped yellowish subretinal lesion nasally to the optic disc (asterisk) and healed lesion along the inferotemporal vascular arcade (arrow) (**A**,**D**). FA of the active tuberculoma shows an early hypofluorescence with late hyperfluorescence, while inactive healed tuberculomas show transmission hyperfluorescence (**B**,**C**). ICGA shows oval hypofluorescent lesions, both in the early and late phases of the exam (**E**,**F**). EDI-OCT active choroidal tuberculoma appears as a hyporeflective lesion involving the choroidal stroma and exhibits increased signal transmission with localized overlying hyperreflective material, indicative of an inflammatory infiltrate, as indicated by the asterisk (**G**); healed choroidal tuberculoma shows alteration of outer retinal layers with increased transmission due to atrophy of the RPE, as indicated by the arrow (**H**).

On eye fundus examination, tuberculoma appears as a solitary, large (up to 14 mm) yellowish round-shaped subretinal lesion, commonly seen at the posterior pole and occasionally in periphery (Figure 3A) [20]. On FA, active tuberculomas show an early hypofluorescence with late hyperfluorescence, while inactive healed tuberculomas show transmission hyperfluorescence (Figure 3B,C) [25]. On ICGA, tuberculoma appears as an oval or round hypofluorescent lesion, both in the early and late phases of the exam (full thickness choroidal granuloma) (Figure 3E,F) [25]. Fundus examination, FA, and ICGA of isolated sarcoid choroidal granuloma are identical to choroidal tuberculoma [26].

On EDI-OCT and SS-OCT images, choroidal tuberculoma appears as a hyporeflective lesion involving the choroidal stroma that exhibits increased signal transmission (Figure 3G,H) [27]. OCT is also helpful in detecting outer retinal changes above the tuberculoma. Salman et al. were the first to describe the “contact sign” as localized hyperreflective material, suggestive of inflammatory infiltrate, in a contact area between the choriocapillaris/RPE complex and the overlying neurosensory retina above the choroidal granuloma associated with surrounding subretinal fluid [28]. Subsequently, Agarwal et al. described the possible fluid accumulation within the photoreceptor layer with a split involving the myoid zone of the photoreceptors [25], termed “bacillary layer detachment” by Mehta et al. in their description of toxoplasma chorioretinitis and pachychoroid disease [29].

Isolated sarcoid choroidal granuloma on EDI-OCT images shares similarities with tuberculoma. It appears as a hyporeflective thickening of the choroid, responsible for a dome-shape appearance of the overlaying retina and RPE. The adjacent choroid has normal thickness and reflectivity. The outer retinal layers overlying the choroidal lesion can be hyperreflective, suggestive of inflammatory infiltrate [30].

Non-invasive follow-up during treatment can be performed with EDI-OCT or SS-OCT to evaluate the improvement of the granuloma (hyporeflective choroidal lesion, subretinal fluid, outer retinal layer changes) or the development of complications (choroidal neovascular membrane or subretinal abscess in cases of TB) [31].

### 2.2. Scleral Inflammatory Lesions

In posterior scleritis, inflammation can spread to the adjacent choroid, causing the following different manifestations of choroidal involvement: (1) increased choroidal thickness, (2) choroidal vasculitis, (3) presentation as a choroidal or subretinal mass in nodular posterior scleritis, and (4) choroidal folds, choroidal effusion, and exudative retinal detachment [32].

#### Nodular Posterior Scleritis

Nodular posterior scleritis is a rare unilateral inflammatory condition affecting only a part of the sclera posterior to the equator, in contrast with diffuse posterior scleritis [33]. Disease onset is generally in the fifth decade of life, with a marked predominance in females. Only a few cases of nodular posterior scleritis have been reported in the literature [34]. The scleral nodule can mimic a choroidal mass, and the rapid recognition of the disease avoids invasive diagnostic surgical procedures, such as chorioretinal biopsy, to exclude neoplastic disorders [35,36,37,38]. A hallmark symptom of nodular posterior scleritis is pain with headache, generally absent in intraocular neoplastic (amelanotic melanoma, choroidal metastasis, choroidal hemangioma, choroidal osteoma) or other inflammatory (choroidal granuloma) conditions [39]. Likewise, the presence of conjunctival injection, chemosis, and limited extraocular motility guides the clinician toward the diagnosis of scleritis instead of neoplastic conditions [40].

**Figure 4:**
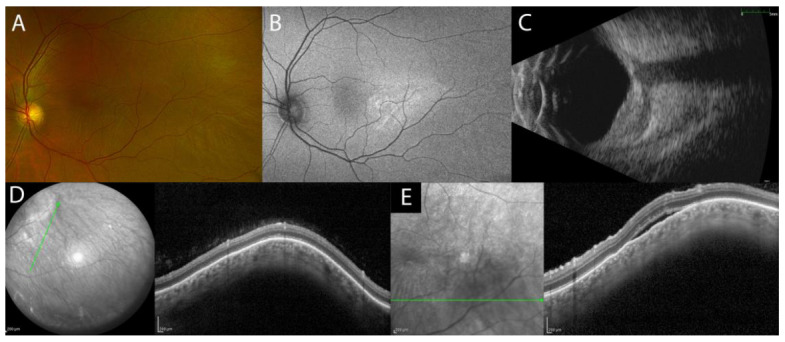
MMI of idiopathic nodular posterior scleritis. UWF fundus image and FAF of amelanotic subretinal mass without lipofuscin or drusen along the inferotemporal vascular arcade and chorioretinal folds (**A**,**B**). B-scan US shows sessile unilobed lesion with high reflectivity and oedema in sub-Tenon’s space (‘T’ sign) (**C**). EDI-OCT shows elevated profile of the retina due to the presence of scleral mass with a normal choroid tissue beneath and focal ERD (**D**,**E**).

On fundus examination, the scleral nodule appears as an isolated amelanotic subretinal mass in the absence of lipofuscin or drusen, with possible associated ERD or retinal/choroidal folds (Figure 4A) [41]. Clinical signs/symptoms (pain) and multimodal imaging (MMI) can help in the differential diagnosis of scleral nodules with intraocular tumors [33].

The combination of FA and ICGA is important to rule out dual circulation, which is a classical finding of choroidal melanoma. The scleral nodule may present with multiple areas of pinpoint leakage and diffuse pooling in the area of the ERD. Occasionally, it is possible to observe optic disc swelling and macular edema. ICGA can show an early hypofluorescence at the location of the ERD and areas of pinpoint hyperfluorescence during late phases due to choroidal inflammation and leakage from the large choroidal vessels [33].

B-scan US, a key exam for the diagnosis and follow-up of the scleral lesion, may show sessile unilobed lesion with high reflectivity and, only in 36% of cases, edema in sub-Tenon’s space (‘T’ sign) (Figure 4C). Computerized tomography (CT) scan and magnetic resonance imaging (MRI) of the orbit with contrast show scleral thickening and are important as complementary exams to rule out choroidal masses [34].

EDI-OCT and SS-OCT can show an elevated profile of the retina due to the subretinal lesion with a normal choroid tissue beneath it (Figure 4D) and occasionally focal or multifocal ERD, retinal/choroidal folds, or macular edema (Figure 4E) (Table 1).

## 3. Neoplastic Lesions

### 3.1. Choroidal Melanoma

Choroidal melanoma is a life-threatening malignancy generally diagnosed on clinical examination by means of fundus examination and ultrasonography. It usually presents as a pigmented (85%), sessile, or dome-shaped lesion characterized by overlying orange pigment and complicated by ERD. US is useful for determining size, thickness, and basal dimension and to exclude extraocular extension. On B-scan US, the dome-shaped profile is the most common, but mushroom/collar stud-shaped is the most classic shape; an irregular shape is uncommon. The tumor typically presents medium-to-low homogeneous internal reflectivity on A-scan US. On MRI, it appears hyperintense on T1—weighted images and hypointense on T2—weighted images.

FA pattern in choroidal melanoma is influenced by tumor size and pigmentation, associated ERD, and hemorrhages. In the early phase, it typically shows irregular hyperfluorescence in cases of RPE/outer retinal atrophy, with variable leakage in late phases. Hypofluorescent spots may be seen due to clumps of orange pigment or pigment mottling. Early frames of ICGA demonstrate hypofluorescence of the lesion and sometimes the intrinsic choroidal vasculature, while in the late phase, different patterns can be observed. Nevertheless, neither FA nor ICGA is considered diagnostic.

**Figure 5:**
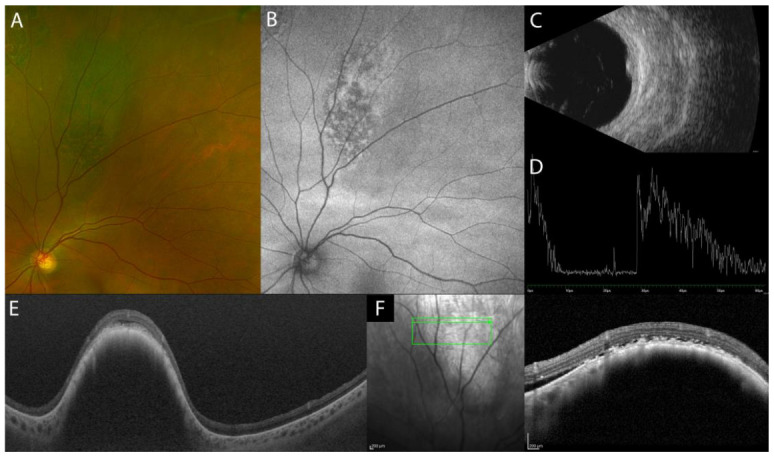
MMI of choroidal melanoma. UWF fundus image of a pigmented lesion with clumped orange pigment (lipofuscin) (**A**). FAF shows a patchy pattern with hyperautofluorescence in the areas of lipofuscin deposit (**B**). US B-scan shows a dome-shaped mass (**C**) with low-to-medium homogeneous internal reflectivity on A-scan (**D**). SS-OCT (**E**) and EDI-OCT (**F**) display dome-shaped choroidal lesion with compression of the choriocapillaris, overlying shaggy photoreceptors, lipofuscin deposition as hyperreflective clumps atop the RPE, and perilesional subretinal fluid.

EDI-OCT of choroidal melanoma generally shows smooth-surface topography with optical shadowing and compression of the overlying choriocapillaris (Figure 5). In a review of EDI-OCT features of small choroidal melanomas, Shields et al. reported that subretinal fluid, lipofuscin deposition, and retinal irregularities, such as shaggy photoreceptors, were common findings. Shaggy photoreceptors overlying choroidal melanoma could represent edematous photoreceptors or macrophages with lipofuscin on the posterior surface of the detached retina and were more frequently found in choroidal melanoma (49%) than in choroidal nevus (0%). Small choroidal melanoma tumor thickness was overestimated by 55% on US compared with EDI-OCT [42].

### 3.2. Choroidal Metastasis

Choroidal metastases are the most frequent form of intraocular malignancy; they generally appear as a small-to-medium size creamy white or pale-yellow mass with poorly defined margins, mean thickness of 3 mm, located posteriorly or at the equator, and are often associated with overlying subretinal fluid.

Choroidal metastases are characterized by a flatter or slightly dome-shaped mass on B-scan US and by medium-to-high nonhomogeneous internal reflectivity on A-scan US. These metastases are mostly multilobular with an irregular surface and may be multifocal and/or bilateral.

FA and ICGA are useful in documenting these lesions. FA typically shows hypofluorescence with mottled hyperfluorescence in correspondence with RPE defects in early phases and irregular hyperfluorescence in the late phases, with possible leakage and pooling in the subretinal space. On ICGA, choroidal metastases appear as well-defined areas of hypofluorescence, with no intrinsic vasculature during all angiogram phases. Minor staining of the lesion may be noticed in the late frames of the angiogram [43,44].

The diagnosis is mainly clinical, but EDI-OCT studies have significantly improved the diagnostic ability to detect small lesions. US overestimates the thickness of small metastatic tumors by a mean of 59% when compared with EDI-OCT. In addition, EDI-OCT is more sensitive than US in the evaluation of recent-onset lesions and response after treatment [45].

**Figure 6:**
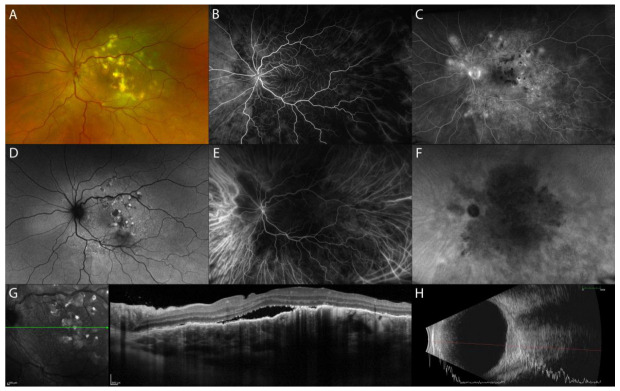
MMI of choroidal metastasis. UWF fundus imaging with creamy white lesion in the posterior pole (**A**). FAF with hypo- and hyperautofluorescence (**D**). FA with hypofluorescence in the early phase (**B**) and pinpoints and leakage areas on the mass in the late phase (**C**). ICGA hypofluorescence in early and late phases (**E**,**F**). EDI-OCT showing “lumpy, bumpy” appearance associated with overlying subretinal fluid (**G**). US flat multilobular mass with medium-to-high internal reflectivity (**H**).

Witkin et al. demonstrated that EDI-OCT can identify subclinical choroidal metastases, barely seen with indirect ophthalmoscopy [46]. The rippled irregular surface appearance, often labeled as a “lumpy bumpy” profile, can be a feature of choroidal metastasis on EDI-OCT. Other lesions with rippled surface include choroidal lymphoma, uveal effusion, hypotony maculopathy, and some forms of uveal inflammatory disease [47]. In choroidal metastasis, EDI-OCT showed a minimally “lumpy bumpy” surface topography with overlying subretinal fluid and shaggy photoreceptors [48]. Low internal optical reflectivity (71%), plateau shape tumor elevation with flat wavy RPE apical surface (75%), overlying choriocapillaris thinning (100%), and partial choroidal shadowing underneath the tumor (71%) were the other common EDI-OCT features in metastatic choroidal tumors (Figure 6) [45].

### 3.3. Choroidal Hemangioma

Choroidal hemangioma is a benign vascular tumor of the choroid, classified as circumscribed or diffuse depending on its distribution and clinical appearance. Choroidal hemangioma may occur sporadically or in combination with Sturge-Weber disease. The circumscribed choroidal hemangioma appears clinically as a round or oval red-orange mass with indistinct margins, classically located posterior to the equator in the macular or paramacular region of the eye. Typically, the diagnosis occurs when the lesion is complicated by intra- or subretinal exudation, resembling central serous chorioretinopathy. Choroidal hemangioma can also simulate an amelanotic choroidal melanoma or metastasis.

**Figure 7:**
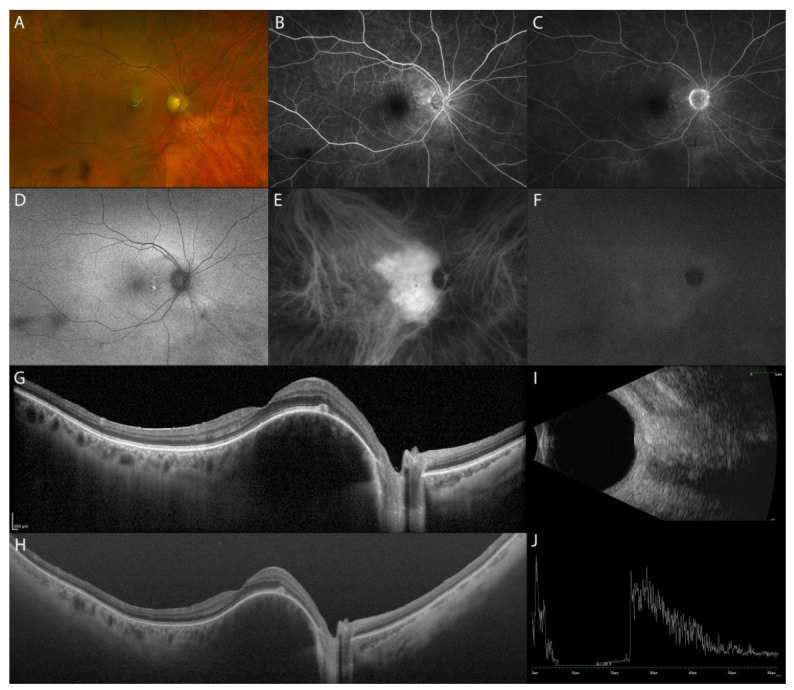
MMI of choroidal hemangioma. UWF fundus image and FAF of choroidal hemangioma in the posterior pole (**A**,**D**). FA showing mild lacy hyperfluorescence (**B**,**C**). ICGA shows early intense hyperfluorescence with washout effect in the late phase (**E**,**F**). EDI-OCT (**G**) and SS-OCT (Optos PLC, Dunfermline, Scotland, UK) (**H**) showing focal thickening of the choroid without compression of the choriocapillaris. US dome-shaped acoustically solid mass similar to the surrounding normal choroid (**I**) with high internal reflectivity (**J**).

FA does not add significant information as it shows varying degrees of hyperfluorescence in all phases. There is early lacy mild hyperfluorescence in the pre-arterial or early arterial phase and diffuse intense hyperfluorescence in the late phase [49]. The ICGA pattern is pathognomonic, showing a rapid lobular filling, with early intense hyperfluorescence in the first minute and a “washout” effect with reduction of the hyperfluorescence in the late phases of the ICGA (30 min) (Figure 7) [49].

On MRI, it appears hyperintense to vitreous on T1 and hyper-isointense on T2 with contrast enhancement [49,50]. On B-scan US, choroidal hemangioma appears as a dome-shaped acoustically solid mass similar to the surrounding normal choroid. On A-scan US, it has high internal reflectivity [51]. Compared to US, tumor thickness measurement by EDI-OCT was 51% less than on US [52].

Rojanaporn et al. reported that on EDI-OCT, choroidal hemangioma shows a smooth, gently sloping anterior contour (100%) with gradual expansion of medium and large size choroidal vessels without compression of the choriocapillaris (100%). The lumen of the choroidal vessels in the choriocapillaris, Haller’s layer, and Sattler’s layer were expanded rather than compressed, as is seen with nevus, melanoma, and metastasis. Other OCT features included partial optical shadowing underneath the hemangioma (90%), subretinal fluid (70%), lipofuscin deposition (10%), intact Bruch’s membrane, and structural abnormalities of outer and inner retinal layers related to chronic subretinal fluid (Figure 7) [52].

### 3.4. Choroidal Lymphoma

Choroidal lymphoma is a rare ocular malignancy, most commonly of B cell origin, that usually appears as amelanotic infiltration of the posterior uvea. It is classified as primary choroidal lymphoma (69%), typically a low grade lymphoma (extranodal marginal zone B-cell lymphoma is the predominant form), or secondary choroidal lymphoma (31%) in the presence of systemic, often high grade lymphoma [47].

Funduscopic findings include multifocal creamy-yellow patches at the level of choroid, ERD, and less frequently, choroidal folds. A detailed clinical examination can be suggestive of choroidal lymphoma, but a definitive diagnosis can only be obtained with biopsy and cytologic evaluation.

The main differential diagnosis includes inflammatory diseases such as birdshot choroidopathy, acute multifocal placoid pigment epitheliopathy, and intraocular tumors, including choroidal metastases, melanoma, and hemangioma. Multifocal yellow-white subretinal infiltrates are also commonly seen in vitreoretinal lymphoma; however, in this condition, the infiltrates are typically located between the RPE and the Bruch’s membrane rather than in the choroid [53].

FA can reveal multiple pinpoint leakages, followed by pooling of the dye as well as optic disc staining. Choroidal infiltration on ICGA is characterized by multifocal sub-millimeter hypofluorescent round areas (300–500 microns diameter) that may resemble a granulomatous uveitis [54,55]. The typical B-scan US appearance is an acoustically hollow thickening of the choroid that, in some cases, can show extrascleral extension [53]. US and MRI show a relatively smooth surface of this malignancy.

EDI-OCT has a sufficiently high resolution (down to the 4 mm level) to demonstrate how the tumor surface varies depending on tumor thickness [56]. Shields et al. described the surface topography of choroidal lymphoma, comparing its features to the ocean surface as calm, rippled, or undulating (seasick). In thin lymphoma infiltration (mean: 1.7 mm), the choroidal surface appeared calm, in medium infiltration (mean: 2.8 mm) it appeared rippled, and in thick infiltration (mean: 4.1 mm) it was undulating or “seasick” [47]. The irregular undulating appearance is most characteristic of choroidal lymphoma but can occasionally be found in choroidal metastasis, choroidal osteoma, and sclerochoroidal calcification, although to a lesser degree [47]. Moreover, in choroidal lymphoma, the choriocapillaris is compressed inward on EDI-OCT images, while medium and large choroidal vessels are generally not detectable.

**Figure 8:**
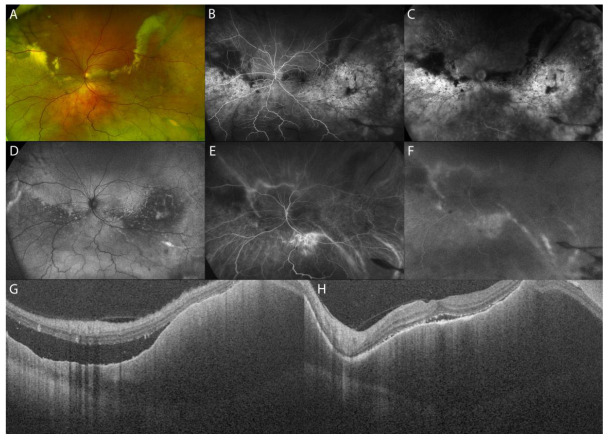
MMI of choroidal lymphoma. UWF fundus image of a patient with choroidal lymphoma and exudative retinal detachment (**A**). FAF (**D**) and FA (**B**,**C**), respectively, show areas of hypoautofluorescence with hyperautofluorescent spots (**D**) and areas of hyperfluorescence with mottled hypofluorescence (**B**,**C**) due to atrophic-pigmentary remodeling of the RPE. ICGA reveals hypofluorescent round areas (**E**,**F**). SS-OCT showing a rippled surface choroidal contour with infiltration and thickening of the choroid, inward compression of the choriocapillaris, and overlying subretinal fluid (**G**,**H**).

Pellegrini et al. analyzed the diagnostic value of combined ICGA and OCT, showing an incongruence between the diffuse choroidal infiltration usually documented on OCT in choroidal lymphoma and its appearance on ICGA, in which multifocal sub-millimeter regions of choroidal hypofluorescence are visible. This diagnostic feature could assist clinicians in differentiating between choroidal lymphoma and other pathological entities (Figure 8) (Table 2) [54].

## 4. Conclusions

The differential diagnosis between inflammatory and neoplastic lesions deforming the choroidal profile can sometimes be challenging. Distinguishing between these conditions is essential for the correct management of the patient and is driven by an accurate clinical examination in association with the support of MMI. The more recently introduced EDI-OCT and SS-OCT imaging modalities provide a deeper penetration and a better resolution of the choroid, resulting in improved characterization of subretinal lesions deforming the choroidal profile (Table 3).

## Figures and Tables

**Table 1:** Imaging findings of inflammatory lesions.

Imaging Findings—Inflammatory Lesions			
Lesion	OCT/EDI-OCT/SS-OCT	FA/ICGA	US
Acute uveitic phase of VKH disease	serous retinal detachment with typical fibrinous septa, diffuse choroidal thickening	FA: pinpoint areas of leakage, subretinal pooling in ERD, hyperfluorescence of optic disc ICGA: hypofluorescent spots	ERD and diffuse, low to medium reflective thickening of the choroid posteriorly
Chronic recurrent uveitic phase of VKH disease	increased choroidal thickness, choroidal localized thickening with ‘choroidal bulging’	FA: hyperfluorescence of optic disc ICGA: hypofluorescent spots	subretinal fibrosis especially in Hispanic patients
Isolated sarcoid choroid granulomas	hyporeflective focal choroidal thickening with increased signal transmission, possible hyperreflectivity of outer retinal layers overlying the choroidal lesion (inflammatory infiltrate)	FA: early hypofluorescence with late hyperfluorescence ICGA: oval/round hypofluorescent lesion both in the early and late phase of the exam	dome-shaped hyperechoic lesion
Tuberculoma	hyporeflective lesion in the choroidal stroma with increased signal transmission, possible “contact sign”	FA: early hypofluorescence with late hyperfluorescence ICGA: oval/round hypofluorescent lesion both in the early and late phase of the exam	dome-shaped hyperechoic lesion
Nodular posterior scleritis	elevated retinal profile, possible: focal or multifocal ERD, retinal/choroidal folds, macular edema	FA: pinpoint leakage, late pooling (ERD), optic disc swelling ICGA: choroidal vasculitis early hypofluorescence with late pinpoint hyperfluorescence	scleral lesion, may show high reflectivity sessile unilobed or dome-shaped lesion edema in sub-Tenon’s space (‘T’ sign)

**Table 2:** Imaging findings of neoplastic lesions.

Imaging Findings—Neoplastic Lesions			
Lesion	OCT/EDI-OCT/SS-OCT	FA/ICGA	US/MRI
Choroidal melanoma	dome-shaped solid choroidal elevation with smooth and regular surface, choriocapillaris compression, possible: SRF, lipofuscin deposition, shaggy photoreceptors	FA: early hyperfluorescence, possible multiple areas of pinpoint leakage ICGA: mixed pattern of fluorescence, blockage of fluorescence in pigmented lesions	B-scan: dome-shaped or mushroom-shaped mass A-scan: medium-to-low homogeneous internal reflectivity
Choroidal metastasis	irregular and lobulated contour, irregular and “lumpy bumpy” anterior surface	FA: hypofluorescent ICGA: hypofluorescent	B-scan: flatter or slightly dome-shaped mass A-scan: medium-to-high nonhomogeneous internal reflectivity
Choroidal hemangioma	smooth, gently sloping anterior contour, choroidal vessels expansion without choriocapillaris compression	FA: early mild hyperfluorescence and late intense hyperfluorescence	B-scan: dome-shaped mass similar to the surrounding normal choroid A-scan: high internal reflectivity
		ICGA: early rapid filling with intense hyperfluorescence and late “washout” effect	MRI: hyperintense to vitreous on T1 and hyper-isointense on T2 with contrast enhancement
Choroidal lymphoma	diffuse choroidal infiltration, choroidal surface depending on tumor thickness: - calm (thin infiltration) - rippled (medium infiltration) - undulating or “seasick (thick infiltration) choriocapillaris compressed inward, medium and large choroidal vessels generally not detectable	FA: multiple pinpoint leakage, dye pooling and optic disc staining ICGA: hypofluorescent multifocal submillimeter round areas	B-scan: might not be detectable, relatively smooth-surface thickening of the choroid, can show extrascleral extension A-scan: acoustically hollow MRI: smooth surface mass within the choroid which shows enhancement on gadolinium contrast

**Table 3:** OCT imaging findings of inflammatory and neoplastic lesions.

OCT Imaging Findings—Inflammatory and Neoplastic Lesions			
Inflammatory Lesion	OCT/EDI-OCT/SS-OCT	Neoplastic Lesion	OCT/EDI-OCT/SS-OCT
Acute VKH disease	diffuse choroidal thickening	Choroidal melanoma	smooth, regular solid choroidal elevation with choriocapillaris compression
Chronic recurrent VKH disease	choroidal localized thickening with ‘choroidal bulging’	Choroidal metastasis	irregular and “lumpy bumpy” anterior surface
Isolated sarcoid choroid granulomas	hyporeflective focal choroidal thickening with increased signal transmission	Choroidal hemangioma	smooth choroidal elevation without choriocapillaris compression, choroidal vessels expansion
Tuberculoma	hyporeflective focal choroidal thickening with increased signal transmission and possible “contact sign”	Choroidal lymphoma	choriocapillaris compressed inward, choroidal surface varies depending on the tumor thickness: calm (thin infiltration), rippled (medium infiltration), undulating or “seasick” (thick infiltration)
Nodular posterior scleritis	elevated retinal profile with normal choroid tissue beneath		

## Data Availability

Not applicable.
